# Individual Differences in Developmental Change: Quantifying the Amplitude and Heterogeneity in Cognitive Change across Old Age

**DOI:** 10.3390/jintelligence6010010

**Published:** 2018-02-28

**Authors:** Nathalie Mella, Delphine Fagot, Olivier Renaud, Matthias Kliegel, Anik de Ribaupierre

**Affiliations:** 1 Cognitive Aging Lab, University of Geneva, 1211 Geneva, Switzerland; Matthias.Kliegel@unige.ch; 2 Center for the Interdisciplinary Study of Gerontology and Vulnerability (CIGEV), University of Geneva, 1211 Geneva, Switzerland; Delphine.Fagot@unige.ch; 3 Methodology and Data Analysis, Section of psychology, University of Geneva, 1211 Geneva, Switzerland; Olivier.renaud@unige.ch

**Keywords:** individual differences, cognitive aging, cognitive heterogeneity, longitudinal method

## Abstract

It is well known that cognitive decline in older adults is of smaller amplitude in longitudinal than in cross-sectional studies. Yet, the measure of interest rests generally with aggregated group data. A focus on individual developmental trajectories is rare, mainly because it is difficult to assess intraindividual change reliably. Individual differences in developmental trajectories may differ quantitatively (e.g., larger or smaller decline) or qualitatively (e.g., decline vs improvement), as well as in the degree of heterogeneity of change across different cognitive domains or different tasks. The present paper aims at exploring, within the Geneva Variability Study, individual change across several cognitive domains in 92 older adults (aged 59–89 years at baseline) over a maximum of seven years and a half. Two novel, complementary methods were used to explore change in cognitive performance while remaining entirely at the intra-individual level. A bootstrap based confidence interval was estimated, for each participant and for each experimental condition, making it possible to define three patterns: stability, increase or decrease in performance. Within-person ANOVAs were also conducted for each individual on all the tasks. Those two methods allowed quantifying the direction, the amplitude and the heterogeneity of change for each individual. Results show that trajectories differed widely among individuals and that decline is far from being the rule.

## 1. Introduction

There is considerable evidence in the literature that the process of aging is associated with cognitive decline [1,2,3,4]. The main bulk of evidence comes from cross-sectional studies, despite the limitations inherent to this design [5,6,7]. In contrast, results from longitudinal studies suggest that cognitive decline is not as pronounced as reported by cross-sectional studies, and that most of cognitive abilities are preserved until the 60s’ or even later [8,9,10]. The difference in results between cross-sectional and longitudinal designs is often attributed to cohort and historical effects or to sampling biases, but most of it is also simply due to interindividual differences in the rate or form of developmental change [11,12]. Only a longitudinal design allows addressing the crucial question of whether individuals differ in their developmental trajectories. 

Studies investigating change in several cognitive domains report interindividual differences in both the rate of change and across domains heterogeneity in the rate of change [13,14,15,16,17,18]. Heterogeneity in change refers to variations in the patterns of change across a number of cognitive abilities. Although often explored by practitioners interested in assessing which competence is affected in a neuropsychological context, this notion has been scarcely explored in research setting. Results from these few studies suggest that change was not necessarily similar in different tasks, either for different individuals or within a given individual. Using latent growth modelling, Johnson et al [13] explored change in several cognitive domains over 36 months in 229 healthy older adults as well as in patients suffering from mild cognitive impairment (MCI). They observed significant change in memory tasks only, but not in processing speed, language, attention and visuo-spatial abilities. Similarly, Mungas et al. [16] reported more age-related change in verbal episodic abilities than in semantic memory or executive function. Go, An and Resnick [19] also explored longitudinal age effects in executive functions and memory in around 150 participants aged over 50 years. The authors report a longitudinal decline over a maximum of 14 years in several abilities, including inhibition, switching, memory, and visuo-spatial abilities, but also stability and/or improvement in abstraction, speed processing, vocabulary, discrimination and chunking. Thus, results from longitudinal studies suggest that aging is not a uniform process, at least in different tasks. We know still less as concerns the homogeneity or heterogeneity of change at the level of an individual.

By extension, very little is known about the degree of heterogeneity of change in the context of neuropsychological diseases. For example, homogeneous decline (i.e., similar across tasks) might reflect a general loss of attentional resources or of mental energy, which could in turn be the reflection of a weakened central nervous system. In contrast, strong heterogeneity in cognitive change might reflect alteration in a single cognitive ability or a subset of cognitive abilities but a good preservation of other cognitive abilities. The degree of heterogeneity in cognitive change may thus be important for diagnostic purposes. However, one needs first to have an overview of how heterogeneous are cognitive changes in healthy aging. All the studies cited above explored change in more or less diverse cognitive functions (executive functions, different forms of memory functions, speed processing, language, etc.) and therefore used different cognitive indexes, such as accuracy, ratios reflecting inhibition capacity or reaction times. Although interesting because of the diversity of cognitive functions that are covered, this may artificially reinforce the across tasks heterogeneity of change. In the present study, we focused on heterogeneity in change in the broad domain of processing speed only, assessed in reaction time tasks of varying complexity.

Most studies interested in age-related change in cognition have remained focused on group performance. Usual analyses allow summarizing general tendencies of a sample, but hides both inter and intraindividual differences that provide information about the diversity in cognitive aging. A number of authors have argued for the necessity to focus analyses on the individual. For instance, Nesselroade and Salthouse, suggest that “the prevailing emphasis on one of the seemingly most fundamental concepts in traditional differential psychology—stability of level of attributes across time—represents an oversimplification that can hinder the search for powerful and general lawful relationships” ([20], p. 49). Moreover, Molenaar has very elegantly demonstrated that a factorial structure applied to a group cannot apply to the individual of this group, thereby invalidating an “ergodicity” hypothesis, that is the relatively frequent hypothesis according to which interindividual variations are informative of intraindividual variations [21,22]. In the same vein, Nesselroade and Molenaar [23] recently suggested that similar results may reflect different meanings for different individuals. Based on a set of simulated data, they show that the presence of subject-specific factor loadings may be detected even though the correlations among the factors are invariant across subjects. In other words, a given psychological structure may differ from one individual to the other, and a same manifest variable may even have a different meaning for different subjects, whereas correlations among the factors are often wrongly taken to indicate that their meaning is the same for all the subjects. All these arguments underline the notion that analyses based on group statistics hinder large interindividual variations and mask the fact that a substantial proportion of individuals composing this group are not represented by these group statistics. Studying age-related change in cognition necessarily requires focusing on the individual if one aims at having a complete picture of cognitive aging, at predicting the diverse trajectories and their cognitive outcomes, or if one wants to ensure of the effectiveness of a given training or intervention. Focusing on the individual when assessing change is thus essential for both research and clinical considerations.

Modern statistical methods, such as latent growth curve modelling, have granted a larger place to individual differences. For instance, they make it possible to assess whether individual differences in change are significant. Yet, they still rely on the group, and do not provide estimates (or better inference) at the level of the individual other than, sometime, a quantitative estimate on how much this individual differs from the adopted model. Importantly, by letting the data of all individuals in the same model, the evaluation of a given individual is automatically influenced by the performance of the other individuals. The effect observed for a given individual might thus be different depending on the sample within which he/she is analyzed. Moreover, all current methods have distributional/parametric assumptions on the interindividual differences, because that is the only way to estimate the parameters of the model. For example, in mixed effect/hierarchical models, the random intercepts or random slopes are supposed to be normally distributed, which means that this model forces the individual differences to have this distributional form. Even if a sample fails to meet this assumption, the model is estimated but the individual effects are biased to match the model characteristics. Thus, the questions of whether change is significant for a given individual, whether it is heterogeneous or homogeneous, whether it goes in the same direction for all individuals remain. Defining the significance of change with aging at the level of an individual is important not only to refine our models of normal aging, to distinguish normal changes from pathological ones, but also for the development and testing of personalized interventions or training aiming at maintaining cognitive abilities in later life.

We are therefore still in need of methods to empirically define and investigate change at the level of the individual, independently of statistics provided by group variations, and remain as long as possible at this level before comparing individuals and groups. Interindividual differences in developmental trajectories have been mostly investigated in terms of correlations analyses (e.g., [24])**,** with a few exceptions [9,25]. For example, exploring cognitive change in a cohort of 1000 elderly catholic clergy members over 15 years, Hayden and collaborators [25] expressed the severity of decline as a function of the standard deviation (SD) proportion relatively to baseline level. They distinguished individuals showing moderate decline (−0.19 SD/year) from those showing severe decline (−0.57 SD/year). It is notable that the majority of participants (65%) were identified as non-declining elderly (−0.04 SD/year). Yet, standard deviation remained in this study a measure established on the group. Based on the Seattle longitudinal study, Schaie [14,15] reported descriptive statistics on individual trajectories in five abilities over 7 years, and showed that only a small percentage of individuals presented an overall significant decline—the significance being defined on the basis of a standard error of measurement—even though the change was significant at the group level. Decline varied across tasks and age groups and was rarely shown by more than a third of the individuals; even for the composite score (i.e., the score marking most decline), this percentage varied from 18% to about 50% (in the oldest group, who aged from 74 to 81 years). Moreover, when decline was observed, it was rarely on more than two tasks, even in the oldest age group. Finally, almost no individual declined over more than two consecutive 7-year intervals across a 28-year period. Schaie’s report clearly contradicts the inference many readers would draw based on group curves. Yet, even in the case of the Seattle data, change is defined based on interindividual differences (standard error of measurement). It has also been proposed to evaluate the clinical significance of change using the reliable change index (RCI), which states the amount of change between two time measurements [26,27]. The RCI is expressed by a ratio of the difference between the time 1 and time 2 related to the standard error of the difference, itself derived from the normative sample. Although clearly focused on individual change, this index depends once again on the sample performance; if the sample size increases (or decreases) conclusion about change in a given individual will also change. It is useful for normative comparison, but does not provide any information concerning the meaningfulness of change relatively to one self’s performance. 

The objective of the present study was to analyze individual trajectories by relying solely on individual data. Two novel and complementary approaches are suggested here to advance this line of research, both of which aim at defining change based on within person data while assuring the reliability of change: (1) a bootstrap based confidence interval approach and (2) an individual analysis of variance approach (for details see below). We demonstrate their use in analyses exploring the magnitude and heterogeneity of individual change with aging across several cognitive measures, including simple reaction times, complex processing speed and inhibition, over a maximum of seven years and a half. Both analyses were made possible because each task contained a large number of trials (from 60 to 144 trials). The bootstrap based confidence interval approach allows defining, for each task and each participant, whether an individual showed a significant decline, stability or improvement over seven years. It thus provides an overview of each participant’s decline/stability/improvement over each of the tasks. The second analysis quantified the degree of significance, for each individual, concerning both the amplitude and heterogeneity of change across all the tasks. This was achieved using individual analyses of variance conducted on the standardized scores by trial, so that performance in all tasks could be compared, for the first and last experimental waves. Results from the Time effect (change between the two time measurements) and for the Time by Task interactions for each individual were used to assess both the amplitude and the significance of heterogeneity of change.

## 2. Materials and Methods 

### 2.1. Participants

Participants for the present analyses come from the Geneva Variability Study (GVS) that started in 2006 as a lifespan cross-sectional study including 557 participants aged 9–89 [28]. This initial wave was followed by a longitudinal follow up in the older adults only (n = 218), who were seen a total of four times at an interval of two and a half years between those four measurement occasions. The present analyses focus on change between the first and last wave of experimentation; this provides the largest interval (approximately seven and a half years), hence the highest chance to observe change. Older adults had been recruited either from the Senior University of Geneva or through newspaper and association advertisements for elderly. All participants were native French speakers or fluent in French and had normal or corrected to normal vision. The study was approved by the ethical committee of the Faculty of Psychology and Educational Sciences of the University of Geneva. All participants gave written informed consent and received a small amount of money as a compensation for their transportation costs.

The initial sample comprised 218 older adults (aged 59–89 years), whose characteristics are displayed in Table 1, and the fourth wave ended with 92 participants (aged 65–93 years). The wave-to-wave attrition rate was between 20% and 29%, which is close to what has been reported in other longitudinal studies of cognitive aging [29,30,31]. As in these studies, attrition was non-random: The older adults who participated in the fourth wave were initially younger and had better baseline levels (on average for the first wave: 67.53 years old; 38.70 Raven Progressive Matrices [32]; 38.49 at the Mill Hill Vocabulary Test [33]) in most cognitive tasks than those who dropped out at earlier steps (a description of the maximal sample for each experimental wave is provided in the Supplementary Material). The present analyses were conducted on the 92 individuals who completed the four waves. 

### 2.2. Experimental Setting

#### 2.2.1. Overview

The GVS included several tasks assessing various cognitive domains, including working memory, simple reaction times, processing speed, interference, fluid and crystallized intelligence tasks as well as questionnaires on health-related issues and life-style [34,35,36]. The tasks were adapted from tasks already used in the cognitive aging literature and shown to present age differences; this is preferable when one adopts multivariate designs. The battery aimed not only at assessing a diverse range of aptitudes, from simple to more complex tasks, but also to make it possible to compare response times and accuracy responses (see Fagot et al., this issue). All experimental tasks were computerized, using a tactile computer screen and administered in a quiet room, in two sessions lasting around two hours each. For comparison purposes, the order of the tasks was kept constant across both participants and experimental waves. The present analyses focused on six reaction time tasks (9 experimental conditions) of different complexity levels: one simple detection task, two choice reaction times tasks, one inhibition task, and two processing speed tasks. All these tasks have the specificity of including a large number of trials; this is a condition for performing both individual bootstrap confidence intervals and individual analyses of variance. 

#### 2.2.2. Tasks

The simple detection task (SDT) consisted in pressing a button when a cross appeared on a screen (see [35,36] for a full description of all the tasks and data pre-processing). In two choice reaction times tasks, participants had to identify the location of the longest line between two (line comparison—LI) or the location of a cross (among six) changing into a square (cross-square—CS). These three tasks contained 120 trials distributed into five blocks.

Processing speed tasks consisted in a letter comparison task (LC) and a digit symbol (DI), both adapted from Salthouse [37] and computerized. In the LC tasks, participants had to determine whether two series of consonants (either 6—LC6 or 9—LC9) were similar or different. There were 60 trials distributed into three blocks for each condition. The DI tasks consisted in identifying whether a symbol-letter association was similar to an initial matrix of nine symbol-letter associations. This task comprised 144 trials distributed into five blocks.

Lastly a Stroop colour task with three conditions, neutral (colored symbols), congruent (e.g., the word blue written in blue) and incongruent (e.g., red written in blue) was given and included 144 trials by condition distributed into 18 blocks.

### 2.3 Analyses

We first investigated change in performance over seven years at the group level and then at the level of the individual. The analyses presented here were based on the mean level of performance (for correct trials only) at the first and fourth wave of testing, computed for each task/condition (some participants did not complete the entire protocol, mainly because of recording errors. It total, 92 participants completed the LI task at baseline and the last experimental wave, 91 the DI and the SDT tasks, 90 the CS task, 88 for the LC task, and 85 the Stroop task (colorblind participants were excluded for this task)) and each individual. Secondly, we investigated how demographic and cognitive measures at baseline were related to both amplitude of cognitive change, and heterogeneity in cognitive change. 

#### 2.3.1 Group Analyses

Paired *t*-test analyses were conducted on mean performance for each task/condition to explore the effect of age on cognitive change at a group level. A Bonferroni correction was applied to correct for multiple testing (9 comparisons). 

#### 2.3.2. Individual Bootstrap Confidence Intervals

To evaluate the change of the individual score distribution between wave 1 and wave 4, we computed confidence intervals (CI) on the difference in the mean score. These CIs were computed for each individual, separately for each task/condition. The choice of T1 for baseline was motivated by exploring change on the longest time-window. However, to ensure that there were not too much practice effects between T1 and T2, we also compared frequency of decline between T1-T2 and T2-T3, assessed with bootstrap confidence interval method (see the Section 2.3.2 for further details on the analyses). Results suggest that there were none-to-very little practice effects (5 conditions out of 9 show a difference of 5 to 10 points indicating more frequent decline for T1-T2, and 4 conditions out of 9 showed a reverse pattern: more frequent decline for T2-T3). A description of these results is given in the Supplementary Material.

CI for the change, measured as the difference in the mean score between wave 1 and 4, were computed for each individual, separately for each task/condition. If the CI contained the value zero, the individual for the given task was declared stable. If the CI was entirely above or below zero, we said there was a decline or an improvement, respectively.

Technically, to prevent the risky effect of possible non Gaussian distribution and serial correlation of the trials on the computation of CIs based on parametric and independence assumptions, we used a stratified block bootstrap method [38]. In the example of a task comprising 120 trials (e.g., LI, SDT, or CS), for each wave, it consisted in cutting the series of 120 trials in 12 pieces of 10 adjacent trials and in resampling with replacement 12 new pieces that formed the new bootstrap sample. This process was repeated 4999 times and the BCa (bias-corrected and accelerated) confidence interval was computed using the boot package from R software (version 3.4.2) [39]. Since we used these confidence intervals to classify changes as stable, declining or improving, and not to make formal significance tests, there was no reason to use the conventional 5% or 1% levels, nor to correct for the multiplicity of CIs/tests. Instead, as in Borella et al. [40], we opted for a 68% confidence interval, which in Gaussian settings would correspond to one standard deviation.

#### 2.3.3. Individual Analyses of Variance

To make them comparable, for each task/condition and each individual, reaction times were first standardized across all trials of the first and the last experimental wave of testing. For example, in the SDT task, data were standardized across 240 trials for each individual, that is, the 120 trials of the first wave and the 120 trials of the fourth wave. Then, we conducted analyses of variance (ANOVAs) with two factors (Time: wave 1 vs. wave 4; Task: nine conditions) for each individual. Results from the main effect of Time addressed the question of a significant overall change in performance over seven years for a given individual, while results from the Time × Task interaction indicated whether an individual showed a similar trend of change in all tasks. Individual effect sizes (partial eta2) were used as a quantification of the degree of across-tasks homogeneity; the lower the eta2, the more homogeneous (similar) is the change across the nine conditions. As concerns the effect of Time, because effect sizes did not provide information concerning the direction of change (improvement or decline), individual regressions were conducted with the same variables and we used the Betas to indicate both the direction and amplitude of change, for each participant. Distributions of both indexes are provided in the Supplementary Material.

#### 2.3.4. Relationships between Cognitive Change and Other Variables

In a subsequent step, we conducted a correlation analysis to explore relationships between, on the one hand, demographic or cognitive variables, and on the other hand, the amplitude and direction of change and the heterogeneity of change with aging. Demographic data included age and education. Cognitive measures included fluid intelligence, vocabulary, as well as both mean level of performance and intraindividual variability in performance, all at wave 1. Intraindividual variability, corrected for both practice effects and individual mean level of performance, was assessed for each task. Intraindividual variability was estimated for each participant by using the individual standard deviation computed on residual scores for each trial, after controlling for the participant’s mean level and the order of trial (see Fagot et al., this issue). Lastly, to simplify the reading and to avoid too many multiple comparisons, both mean and intraindividual variability were pooled in three cognitive domains: simple reaction times (average of CS, LI, SDT), complex processing speed tasks (average of DI, LC6, LC9), interference (average of STn, STi, STc). These tasks were pooled based on a priori hypothesis of similar cognitive complexity, which was confirmed by subsequent factor analyses. 

## 3. Results

### 3.1. Group Analyses 

**Figure 1:**
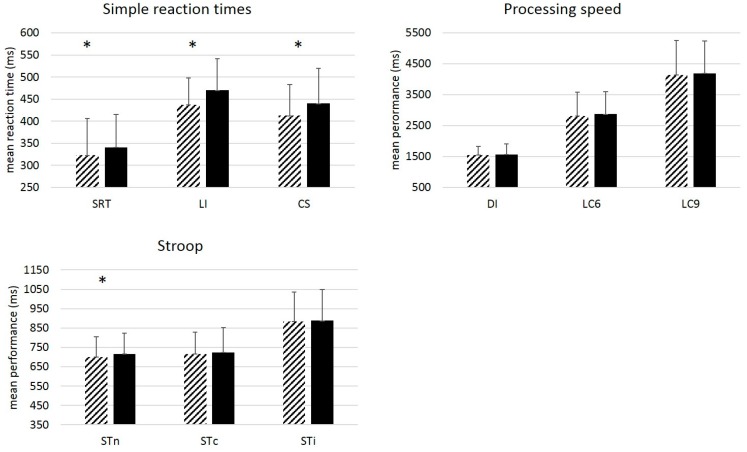
Mean performance (ms) by Task and Wave. Simple RT tasks (SDT, LI, CS), Processing speed tasks (DI, LC6, LC9) and Stroop task (STn =neutral condition, STc = congruent condition, STi = incongruent condition). Dashed bars: Wave 1. Plain black bars: Wave 4. Paired *t*-tests showed significant change in performance for simple tasks only. Error-bars represent standard deviations. * = *p* < 0.005 (Bonferroni correction).

Results (Figure 1) from paired *t*-tests showed a significant slowing of performance over 7 years for the SDT task, t(90) = −3.07, *p* = 0.003, η^2^ = 0.10, for the LI and the CS tasks, t(88) = −5.89, *p* < 0.001, η^2^ = 0.29, t(89) = −5.75, *p* < 0.001, η^2^ = 0.27, respectively, as well as for the neutral condition of the Stroop task, t(84) = −3.53, *p* < 0.001, η^2^ = 0.13. No significant change was observed for the other conditions. 

### 3.2. Individual Analyses 

#### Bootstrap Confidence Intervals

**Figure 2:**
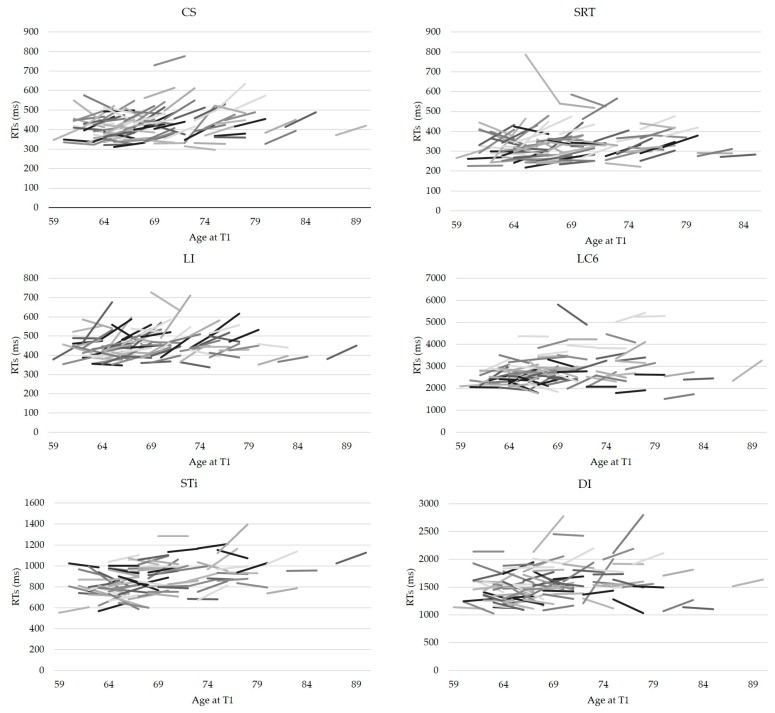
Intraindividual change by Age and Condition (six conditions out of nine). Each segment represents performance of a given individual at baseline and at the fourth experimental wave. Age is indicated at performance at baseline; each segment corresponds to approximately 7–8 years.

**Figure 3:**
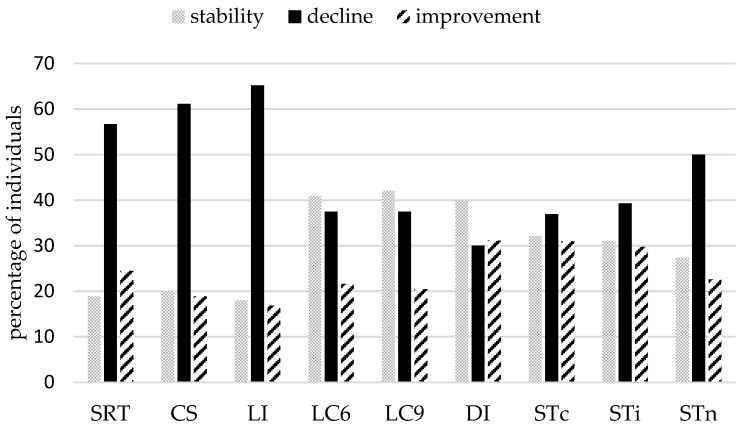
Bootstrap analyses: Percentage of individuals by task/condition and by pattern of change. Bars represent the percentage of individuals showing significant decline over seven years (black bars), significant improvement (dashed bars) and no significant change (grey bars).

Inspecting individual trajectories underlying these group effects suggested, as predicted, enormous inter-individual variability and heterogeneity of changes (see Figure 2). Indeed, bootstrap confidence intervals showed that the three possible patterns of change—decline, improvement and stability—were present in all conditions. However, a larger proportion of individuals who declined was observed for simple reaction time tasks: More than half of the sample showed a slowing down in SDT (57%), LI (65%), CS (61%), and to a lesser degree STn (50%), the neutral condition of the Stroop task. These results join in with the group analyses, that showed a significant change at the group level in these tasks. For all other conditions, the three patterns were well represented (see Figure 3), with a substantial part of the sample showing improved performance after seven years (between 20% and 31% according to the condition). 

**Figure 4:**
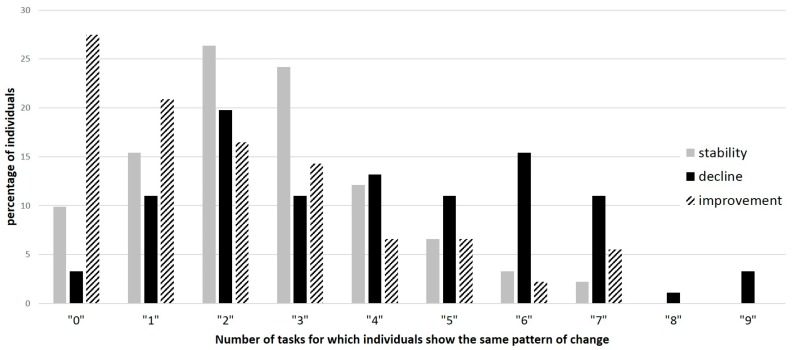
Heterogeneity in the pattern of change across all conditions: decline (black bars), improvement (dashed bars) and stability (grey bars). For example, 27% never presented a significant improvement, and 16% of individuals showed decline in six conditions.

We further analyzed homogeneity in the patterns of change, i.e., whether individuals showing a given pattern of change in one condition also showed the same pattern in the other conditions. Results showed quite heterogeneous patterns of change across conditions (see Figure 4). For example, there was no individual showing stability or improvement in all conditions, and very few individuals (less than 10%) improved significantly in more than three conditions; roughly 25% showed no significant improvement at all (Figure 3, improvement in 0 task). Stability was rarely observed in more than four tasks (less than 10% for more tasks). Decline was somewhat more homogeneous. 

### 3.3. Individual Analyses of Variance

Individual ANOVAs were conducted on 83 individuals only, as nine older adults did not complete all the trials in all the conditions at wave one and/or wave four and these analyses require a fully complete data set for each individual. Results showed a significant main effect of Time in 65 out of 83 older adults. Exploration of the direction of change revealed a global decline in 48 individuals and a global improvement in 17 individuals. A significant Time × Task interaction was observed in the quasi-totality of our sample (*ps* < 0.05), with the exception of four individuals who showed a homogenous trend of change across all the tasks. All the rest of the sample showed heterogeneous trends of change across the tasks.

### 3.4. Relationships between Change and Other Variables

Correlations were computed between the pattern of change (amplitude—beta; heterogeneity—partial eta2) and the cognitive variables administered at baseline. 

Results showed a significant correlation between the heterogeneity in cognitive change and the initial level of intraindividual variability in complex processing speed tasks, *r* = −0.38, *p* < 0.001 (see Table 2, still significant after a Bonferroni correction). This negative correlation indicates that a lower level of variability at baseline was associated with a more heterogeneous subsequent change in performance. A negative correlation was also observed between age at baseline and amplitude of change, *r* = −0.27, *p* = 0.016, indicating that older participants at baseline were those showing a more pronounced subsequent decline. However, significance of this test did not stand the Bonferroni correction. 

## 4. Discussion

The present study aimed at examining individual change in a number of cognitive tasks of varying complexity, by defining change independently of the sample characteristics. We explored two statistical approaches allowing quantifying both amplitude/direction and heterogeneity in individual change. The use of those statistical tools was possible because our data set included tasks with a large number of trials in each condition. Results from bootstrap confidence intervals showed a large heterogeneity in the patterns of change—decline, stability or improvement—across both tasks and individuals; that is, most individuals did present a different pattern of change across tasks, and interindividual differences were also large. Individual analyses of variance revealed that this heterogeneity was significant for almost all participants. Lastly, correlational analyses showed that the degree of heterogeneity of change was negatively related to intraindividual variability at baseline in processing speed tasks.

The first notable result concerns the comparison between group-based and individual-based change analyses. Group-based analyses (*t*-tests) showed that, overall, our sample of older adults became significantly slower in simple reaction tasks—SDT, LI, CS and the neutral condition of the Stroop task—but not in more complex conditions. Looking at individual confidence interval results for these four tasks/condition, we also observed a larger proportion of individuals showing decline (between 50% and 65 %). These results are thus consistent with each other. Yet, the proportion of participants showing stability or improvement was large (between 35% and 50%). Significant decline at the group level was thus driven by individual decline in less than two thirds of the sample, suggesting that at least 35 % of individuals were not well represented by the group tendency. Exploration of the more complex cognitive tasks revealed that the three patterns of change were more equally represented, suggesting that more complex processing speed either is subject to more inter-individual variations or possibly declines later with aging. These results make echo to Schaie’s observation that longitudinal change in aging is driven by change in only a small to moderate proportion of the sample [6,18]. They further suggest that most of aging studies, even those using a longitudinal design, tend to overestimate age-related cognitive changes. Hence the need to extend analyses beyond change at the group level.

An additional argument to explore change at the individual level is the observation of considerable intraindividual heterogeneity in the patterns of change. Intraindividual analyses of variance indeed suggest that the quasi-totality of our sample showed significant heterogeneous change across tasks, even though the tasks are all tapping processing speed. A closer examination of each task, based on the confidence intervals, shows that only a maximum of 16% of individuals demonstrated a similar pattern of change in more than four tasks/conditions out of nine. When an individual declines in a task, the probability of observing the same trend in another task is rather low. These results, together with those exploring group effects, strengthen previous observations that cognitive abilities do not have the same developmental trajectory across the lifespan [13,16,17,19,41]. They also demonstrate not only that differences in developmental trajectories vary across individuals but that such heterogeneity is actually the rule in normal aging. This argues in favor of dissociating factors that may differentially affect cognitive aging in individuals. 

Questions remain concerning the meaningfulness of such heterogeneity: Is a more homogeneous change indicative of later pathological processes or, on the contrary, indicative of a better cognitive development than heterogeneous change? Correlational results indicated no relationships with the amplitude/direction of change, showing that a more homogeneous change does not necessarily go in the direction of cognitive decline. Interestingly, higher within-task intraindividual variability at baseline was significantly associated to a more homogeneous subsequent evolution of cognitive abilities; that is, individuals who presented a larger trial-to-trial within-task variability (averaged across tasks) were more uniform in their change. Within-task intraindividual variability in reaction times in aging has been related to negative subsequent outcomes, including change in cognitive status and attrition [42]. In Bielak et al. study, intraindividual standard deviation (iSDs) in complex tasks provided stronger prediction of change in cognitive status than iSDs in simple tasks, suggesting a greater age-related sensitivity for complex tasks. In the light of these results, one may hypothesize that changing in a more homogeneous way may constitute an early marker of later cognitive troubles. However, in Bielak et al. study, cognitive decline was measured with one task only; it is therefore not possible to extend their conclusion to the homogeneous or heterogeneous character of change. It is also interesting to note that we did not observe a significant relationship between either amplitude or heterogeneity of change, on the one hand, and demographic variables, such as age or education level, on the other hand, nor with the initial mean performance in the diverse cognitive abilities at baseline. Therefore, even though associated with inconsistency in complex processing speed tasks (iSD), reflecting a negative cognitive functioning, homogeneity in change did not depend upon the initial general level of cognitive performance. In order to have more indication on the relationships between heterogeneity and the direction of change, we conducted an ad-hoc exploration of how the patterns of change (decline, improvement, stability) were reparsed in individuals showing extreme homo/heterogeneity, using the first and last terciles. We observe no noticeable differences when looking at the number of “decline” (all tasks being considered): 125 in the group showing the more homogenous change against 113 in the group showing the more heterogeneous change. However, when looking at patterns of “improvement”, data show a remarkable difference between the two groups: 26 in the group showing the more homogenous change against 78 in the group showing the more heterogeneous change. This observation suggests that more positive outcomes are associated to heterogeneous change in our sample. At the present stage, we can only speculate on possible mechanisms underlying homogeneity/heterogeneity in cognitive change. Our results suggest that a more heterogeneous change may have more favorable outcomes than homogeneous change. Futures studies focusing not only on quantitative but also qualitative individual differences in change, as well as on association with other variables, would help building stronger hypotheses. 

The two methods presented here may offer potential tools for practitioners. Bootstrap confidence intervals at the level of the individual may notably be useful to assess the efficiency of cognitive training or remediation and constitutes an interesting alternative to the more frequently used RCI. Provided the number of trials is sufficient, they make it possible to assess individual change in performance, without the inconvenience of having to compare with change in a normative sample, subject to interindividual variability issues. Unlike the RCI, conclusions based on the bootstrap confidence interval would not vary if a given individual was studied or if participants were added or removed from the sample. Our method offers a reliable tool to draw conclusions solely based on individual performance. The index of heterogeneity/homogeneity of change in diverse cognitive abilities may also be a meaningful tool for practitioners. The design of the present study does not allow exploring the becoming of individuals showing a more or less homogeneous decline across tasks and further experiments are necessary to refine our understanding of interindividual differences in cognitive change in both normal and pathological aging. Our results open interesting directions of research concerning heterogeneity in cognitive change. A question concerns the meaning of heterogeneity as a function of the cognitive domains. We conducted analyses within the broad domain of speed processing, but we might observe different results in another cognitive domain (e.g., memory). Future studies should provide further data to have a more complete view of how heterogeneous cognitive change is in normal aging. 

This study presents a number of limitations that have to be underlined. First, the sample is composed of older adults coming on their own at university to undergo a battery of tests. Our participants were then cognitively well preserved and reflect only part of the population of healthy older adults. Note that this is the case of a large number of cognitive aging studies. Second, the sample is rather small, particularly when the focus is placed on individual differences. Future studies should be conducted at larger scales. Third, the proposed analyses, being based on merely two time measures can only assess change in the cognitive abilities and cannot educate on the trajectories of change. It is however highly probable that not all individuals experience cognitive decline in the same manner. As mentioned, results from confidence-based analyses of change for intermediate experimental waves suggest a huge interindividual variability in the pattern of change over time, as well as across-tasks intraindividual variability in the pattern of change (change may be linear for a given task but not for another one). Further analyses of interindividual differences in the patterns of change were beyond the scope of the present study, but would undoubtedly be needed to fully understand individual developmental trajectories in healthy aging and the characteristics of trajectories that might be related to unhealthy aging.

## 5. Conclusions

To conclude, this study constitutes, to our knowledge, a first attempt to explore a reliable change (amplitude and heterogeneity) at the level of the individual, with no influence of the sample size or of the composition of the group. Although still essentially descriptive, it provides a general picture of the large heterogeneity of cognitive change across and within individuals, which is much larger than what could be expected based on the extant literature. Reciprocally, it points to the necessity to analyze change at the level of the individual. One can also wonder whether a more homogeneous change under the form of a cognitive decline should call for more attention on the clinicians’ part. The methodology proposed here brings a novel way of investigating within individual changes and offers new considerations on this increasingly crucial research question. 

## Figures and Tables

**Table 1:** Participants’ characteristics at baseline and at the last experimental waves (N = 92).

Time of Measurement	Age	Fluid Intelligence ^1^	Vocabulary ^2^
	M (SD)	M (SD)	M (SD)
Baseline	67.54 (5.51)	38.71 (8.02)	38.49 (4.27)
Last experimental wave	73.79 (5.59)	39.68 (8.37)	38.67 (4.16)

^1^ Raven Progressive Matrices; ^2^ Mill Hill Vocabulary Test.

**Table 2:** Correlations between demographic and cognitive variables and amplitude and heterogeneity of cognitive change.

	Amplitude of Change (Beta)	Heterogeneity in Change (Partial eta2)
Age	−0.27	−0.17
Education ^1^	0.22	−0.01
Simple RT_mean	0.15	0.10
Processing speed_mean	0.02	−0.21
Inhibition_mean	0.14	−0.13
Simple RT_IIV	0.09	−0.18
Processing speed_IIV	0.11	−0.38 *
Inhibition_IIV	0.04	−0.11
Raven PM	0.15	0.15
Mill Hill	0.20	0.14
Heterogeneity in change	−0.01	

Note: coefficients of correlation between, on the one hand, the amplitude of change and heterogeneity in cognitive change and, on the other hand, demographic and cognitive variables. ^1^ Number of education years. * *p* < 0.002; Bonferroni corrected for 21 comparisons.

## Supplementary Materials

The following are available online at http://www.mdpi.com/2079-3200/6/1/10/s1, Figure S1: Bootstrap analyses: Percentage of individuals by task/condition and by pattern of change, Table S1: Participants' characteristics at baseline and at each experimental wave
