# Supplementary material for: Individual Differences in Developmental Change: Quantifying the Amplitude and Heterogeneity in Cognitive Change across Old Age
Source: J Intell. 2018 Feb 28;6(1):10. doi: 10.3390/jintelligence6010010 (PMC6480728; doi:10.3390/jintelligence6010010)
Supplement: Supplementary file 1 [file jintelligence-06-00010-s001.pdf]

Journal of Intelligence

Supplementary Material

Individual differences in developmental change: Quantifying the amplitude and heterogeneity in cognitive change across old age

Nathalie Mella, Delphine Fagot, Olivier Renaud, Matthias Kliegel and Anik de Ribaupierre

A Participants’ characteristics at each of the experimental waves

Table S1. Participants' characteristics at baseline and at each experimental wave

	Sex	Age	Fluid intelligence [1]	Vocabulary [2]
	F/M	M (SD)	M (SD)	M (SD)
Wave 1	165/54	70.10(6.78)	36.47(9.12)	37.73(4.64)
Wave 2	132/42	71.59(6.45)	37.26(8.62)	37.80(4.77)
Wave 3	96/34	72.86(6.10)	38.93(8.92)	38.27(5.01)
Wave 4	73/19	73.79(5.59)	39.68(8.37)	38.67(4.16)

[1]Raven Progressive Matrices; [2] Mill Hill Vocabulary Test

B Distribution of the Partial eta squared and Betas parameters

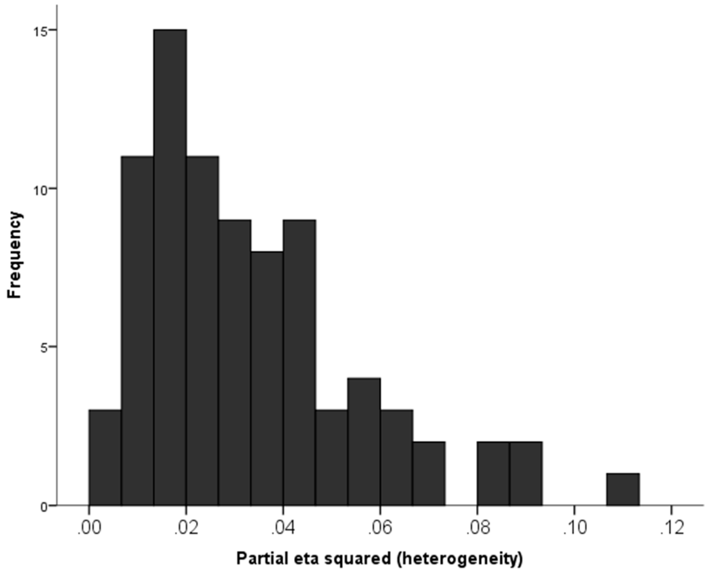

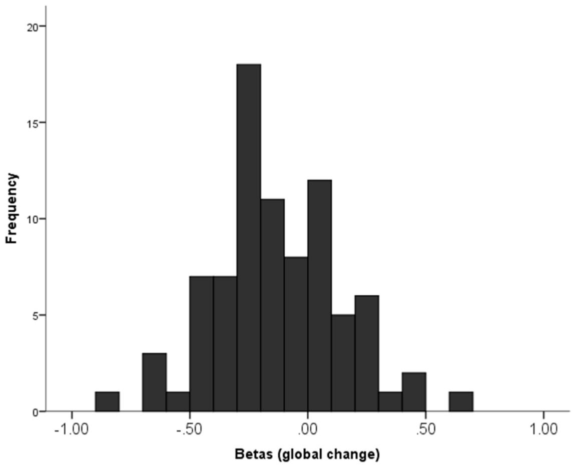

C Results from bootstrap confidence interval concerning intermediate experimental waves (second and third experimental waves) for each task.

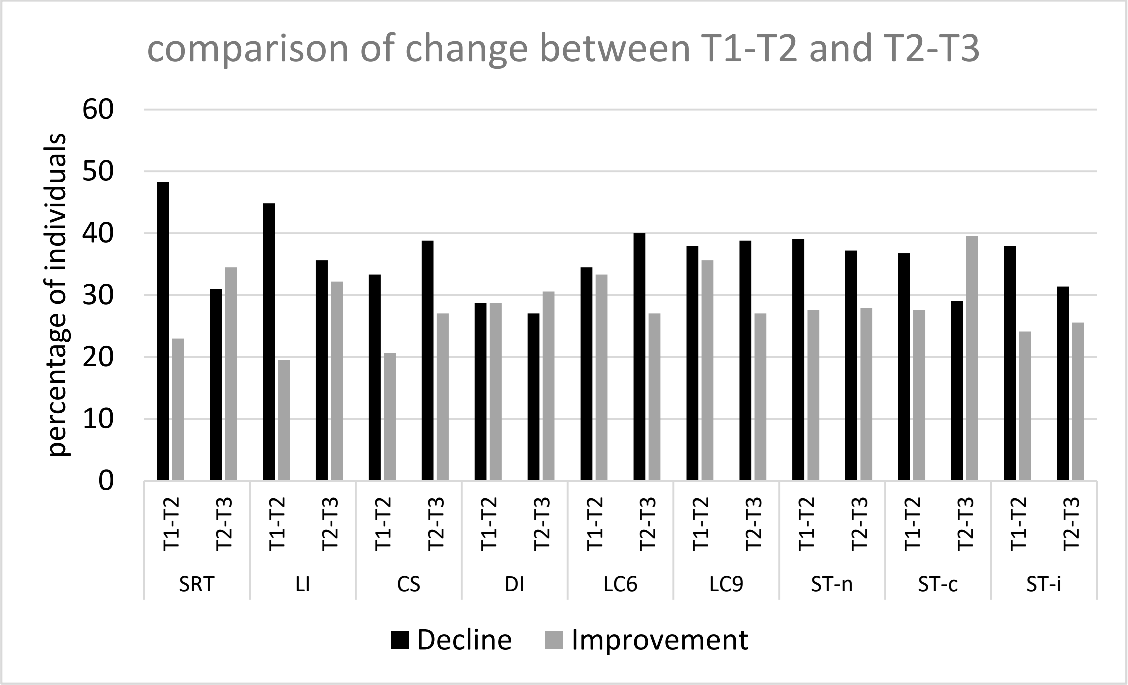

Figure S1. Bootstrap analyses: Percentage of individuals by task/condition and by pattern of change. Bars represent the percentage of individuals showing significant decline (black bars) and significant (grey bars) over seven years. Results compare these patterns between T1 and T2 and between T2 and T3. Results show more frequent decline for T1-T2 than for T2-T3 in 5 out 9 conditions (with a difference going from 5.01 to 10.64 points), while decline was more frequent in T2-T3 than in T1-T2 in the 4 remaining conditions (with a difference ranging from 1.48 to 5.11 points). This observation suggest that there is very little if no retest effect between T1 and T2.
